# Engineered Neutral Phosphorous Dendrimers Protect Mouse Cortical Neurons and Brain Organoids from Excitotoxic Death

**DOI:** 10.3390/ijms23084391

**Published:** 2022-04-15

**Authors:** Inmaculada Posadas, Laura Romero-Castillo, Rosa-Anna Ronca, Andrii Karpus, Serge Mignani, Jean-Pierre Majoral, Mariángeles Muñoz-Fernández, Valentín Ceña

**Affiliations:** 1 Unidad Asociada Neurodeath, Facultad de Medicina, Universidad de Castilla-La Mancha, 02006 Albacete, Spain; inmaculada.posadas@uclm.es (I.P.); laura.romero.castillo@ki.se (L.R.-C.); ros-anna88@hotmail.it (R.-A.R.); 2 Centro de Investigación Biomédica en Red (CIBER), Instituto de Salud Carlos III (ISCIII), 20029 Madrid, Spain; 3 Laboratoire de Chimie de Coordination (LCC), Centre National de la Recherche Scientifique (CNRS), 31400 Toulouse, France; andrii.karpus@lcc-toulouse.fr (A.K.); jean-pierre.majoral@lcc-toulouse.fr (J.-P.M.); 4 Department Medicinal Chemistry, Université Paris Descartes, 75006 Paris, France; serge_mignani@orange.fr; 5 Centro de Química da Madeira, Universidade da Madeira, 9000-072 Funchal, Portugal; 6 Department of Immunology, Hospital General Universitario Gregorio Marañón, 28007 Madrid, Spain; mmunoz.hgugm@gmail.com

**Keywords:** phosphorous dendrimers, excitotoxicity, cortical neurons, brain organoids, neuroprotection, mitochondria

## Abstract

Nanoparticles are playing an increasing role in biomedical applications. Excitotoxicity plays a significant role in the pathophysiology of neurodegenerative diseases, such as Alzheimer’s or Parkinson’s disease. Glutamate ionotropic receptors, mainly those activated by N-methyl-D-aspartate (NMDA), play a key role in excitotoxic death by increasing intraneuronal calcium levels; triggering mitochondrial potential collapse; increasing free radicals; activating caspases 3, 9, and 12; and inducing endoplasmic reticulum stress. Neutral phosphorous dendrimers, acting intracellularly, have neuroprotective actions by interfering with NMDA-mediated excitotoxic mechanisms in rat cortical neurons. In addition, phosphorous dendrimers can access neurons inside human brain organoids, complex tridimensional structures that replicate a significant number of properties of the human brain, to interfere with NMDA-induced mechanisms of neuronal death. Phosphorous dendrimers are one of the few nanoparticles able to gain access to the inside of neurons, both in primary cultures and in brain organoids, and to exert pharmacological actions by themselves.

## 1. Introduction

In nanomedicine, engineered nanoparticles (NPs) have sparked a rapidly growing interest for diverse biomedical applications, including their use as delivery platforms (nanocarriers) for drugs or nucleic acids [1]. NPs have revolutionized research and changed our current concept of therapeutics and diagnostics. Some of these NPs have been used in clinical trials to deliver therapeutic compounds to treat diverse diseases, such as hereditary transthyretin amyloidosis [2] and acute intermittent porphyria [3]. In addition, some of them are key elements to vehiculate mRNA-based COVID-19 vaccines [4]. However, there is a group of diseases, neurodegenerative diseases, where NPs are still far from contributing to an efficient therapeutic approach. Neurodegenerative diseases, including, among others, Alzheimer’s and Parkinson’s disease, have an increasing incidence in the population due to increased life expectation [5]. This lack of NP efficiency can be due to the difficulty NPs face in crossing the blood–brain barrier (BBB) that stands between the blood stream and the brain parenchyma, strongly limiting the passage of foreign substances, such as drugs and NPs, into the brain [6] and/or contributing to the difficulty for NPs to enter non-dividing cells, such as neurons, once they are inside the brain.

Different NP types have been proposed for a broad range of biomedical applications [7,8]. Dendrimers are well-defined, nano-sized, homogeneous, monodisperse structures consisting of (1) a central core that allows various branch-type linkages, which are composed of repeated units leading to a series of radially concentric layers named generations (G), and (2) terminal functional groups that facilitate interactions with molecules of biological interests [9]. Dendrimers allow more specific drug targeting and delivery of small drugs [10] or siRNA [11] to different cell types. One specific class of dendrimers, phosphorous dendrimers, has shown several noteworthy biological activities, including anti-inflammatory [12], anti-prion [13], and anti-cancer [14] effects both in vitro and in vivo.

Organoids are tridimensional structures that can be generated from stem cells or from induced pluripotent progenitor cells [15]. Organoids are not complete organs but representative parts of the original organs reproducing some of their structural, molecular, and functional characteristics [16,17]. Brain organoids have a 3D structure much closer to the human brain than that found in laboratory animals, allowing more precise and reliable studies than those involving laboratory animals or primary neuronal cultures [18]. This similarity, together with the human origin of the cells used to generate them, makes brain organoids suitable models for reproducing the behavior of the human brain in models of human pathology, such as neurodegenerative diseases [19], providing results that can be less prone to fail in future clinical trials.

Glutamate is the main excitatory neurotransmitter in the central nervous system (CNS) playing a central role in neuronal plasticity, learning, and memory [20]. However, when extracellular glutamate concentration increases to levels above physiological levels, it causes aberrant synaptic signaling, which leads to excitotoxicity and neuronal death [21]. Excitotoxicity is a common mechanism causing neuronal damage in different human diseases, such as stroke, nervous system trauma, epilepsy, and chronic neurodegenerative disorders, including Parkinson’s and Alzheimer’s disease [22]. Excitotoxicity causes neuronal death by over-stimulation of ionotropic glutamate receptors, the N-methyl-D-aspartate receptor (NMDAR) being the main glutamate receptor subtype involved in excitotoxicity [23]. NMDAR over-stimulation increases intracellular Ca^2+^ concentrations ([Ca^2+^]_i_), leading to mitochondrial dysfunction, which is characterized by a decrease in mitochondrial potential (Ψm); increase in reactive oxygen species (ROS) production; electron transport chain dysfunction; decrease in ATP production; and release of pro-apoptotic factors, including cytochrome c and apoptotic protease activating factor 1 (Apaf-1), leading to sequential activation of caspases 9 and 3 [24].

In addition to high-Ca^2+^-mediated mitochondrial mechanisms, excitotoxicity triggers endoplasmic reticulum (ER)–Ca^2+^ homeostasis disruption [25], which can contribute to neuronal cell death through two main pathways. On the one hand, it can induce an apoptosis crosstalk between both organelles followed by the mitochondria-mediated toxicity events mentioned above [26]. On the other hand, impairment of ER functioning triggers the activation of the unfolded protein response (UPR), leading to a shutdown of translation and an over-expression of ER-stress-related proteins [27]. Prolonged UPR activation also induces cell death by activating, among others, proteins-like eukaryotic initiation factor 2α (eIF2α), immunoglobulin heavy-chain-binding protein (GRP78/Bip), activator transcription factor 4 (ATF4), C/EBP homologous protein (CHOP), and caspase 12, which are major components of ER-stress-induced apoptosis [28]. It is important to note that the inhibition of Ca^2+^ release from the ER protects cortical neurons against NMDA-induced excitotoxicity by attenuating both mitochondrial damage and ER stress [29].

In addition to an increase in [Ca^2+^]_i_, mitochondrial disfunction, and ER stress, excitotoxicity generates an inflammatory response [30]. Thus, we decided to study the effect of neutral phosphorous dendrimers of generations 3 and 4 (Figure S1) that have previously displayed a marked anti-inflammatory action [12], both in vitro and in vivo, on a well-established in vitro model of excitotoxicity such as NMDA-mediated toxicity both in primary mouse cortical neurons [21] and in human brain organoids.

We found that both phosphorous dendrimer generations markedly decreased NMDA-mediated excitotoxicity in primary cortical neurons by an action downstream from NMDA-mediated Ca^2+^ entry that involves decreased production of mitochondrial ROS, ER stress, and UPR responses. The dendrimers also showed a marked neuroprotective action in NMDA-treated human brain organoids by decreasing NMDA-induced caspase 3, 9, and 12 activation as well as neuronal death. These data strongly suggest that neutral phosphorous dendrimers can penetrate not only neurons in culture but also a 3D structure, such as the brain organoid, and that this family of dendrimers might represent a useful scaffold to design new NP-based therapeutic agents for treating neurodegenerative diseases.

## 2. Results

### 2.1. Effect of Phosphorous Dendrimers on Excitotoxicity

**Figure 1:**
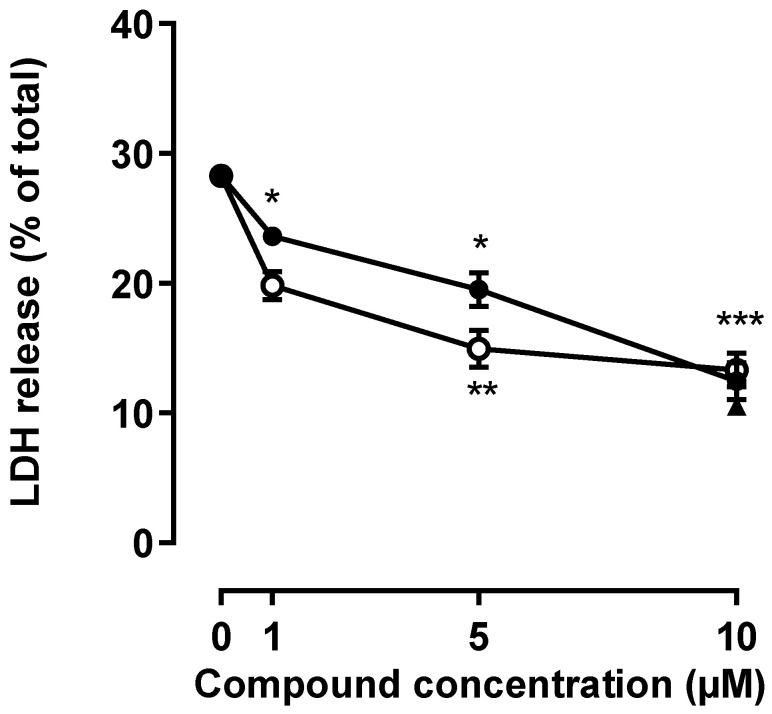
Phosphorous dendrimers prevent NMDA-mediated excitotoxicity. Neurons were treated with the indicated concentration of phosphorous dendrimers G3 (open circles), G4 (closed circles), or MnTBAP (10 µM; closed triangle) for 1 h; and then NMDA (150 µM) was added and incubation continued for another 24 h. LDH activity was determined as indicated in the Materials and Methods section. Zero concentration represents net (stimulated–basal) NMDA-induced LDH release in the absence of any other treatment. The data represent the mean ± the s.e.m. of 6 to 12 independent experiments. * *p* < 0.05, ** *p* < 0.01, and *** *p* < 0.001 when compared to NMDA-treated cells.

To study the effect of G3 and G4 phosphorous dendrimers on NMDA-induced neuronal death, mouse cortical neurons were treated with vehicle or NMDA (150 µM; 24 h) in the presence or absence of phosphorous dendrimers (1 to 10 µM). As expected, NMDA induced a significant increase in the LDH released, which amounted to about 37% of the total LDH content. Both phosphorous dendrimer generations, G3 and G4, markedly decreased NMDA-induced neuronal death in a concentration-dependent manner, G3 being slightly more potent than G4. At the maximal concentration studied (10 µM), both phosphorous dendrimer generations almost halved NMDA-induced neuronal death (Figure 1).

### 2.2. Phosphorous Dendrimers Lack Any Effect on an NMDA-Induced Increase in [Ca^2+^]_i_

**Figure 2:**
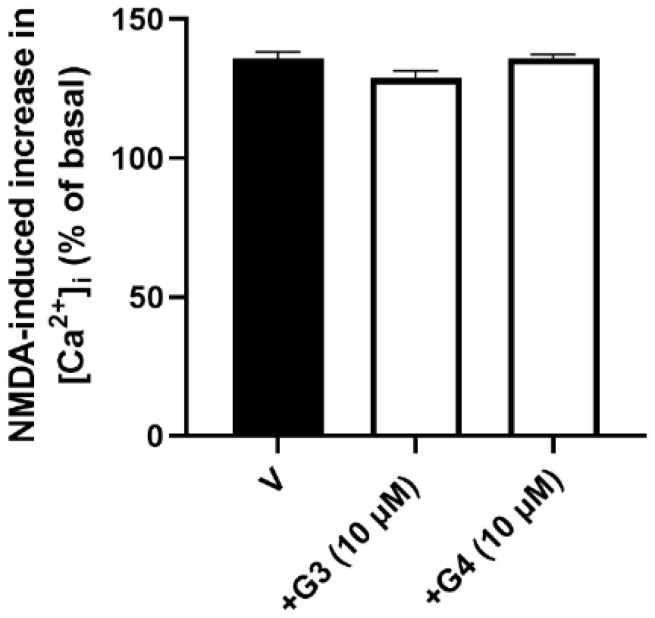
Effect of phosphorous dendrimers on NMDA-induced increase in [Ca^2+^]_i_. Neurons were treated (10 µM; 1 h) with G3 or G4 phosphorous dendrimers. Then, the neurons were incubated with Fura-2 and exposed to NMDA (150 µM) in the absence (V) and presence of dendrimers. Data represent the percentage over basal line (100%) of the ratio of fluorescence taken as an index of [Ca^2+^]_i_, as indicated in the Materials and Methods section. The data are expressed as the mean + the s.e.m. of 30 to 40 neurons obtained from three independent experiments.

Phosphorous dendrimers might decrease NMDA-mediated excitotoxic neuronal death by interfering with NMDA-induced increase in [Ca^2+^]_i_, which is related to neuronal death [31]. Treatment of cortical neurons with NMDA (150 µM) induced almost a 40% increase in [Ca^2+^]_i_, which was not modified by the presence of phosphorous dendrimers (Figure 2 and Figure S2), suggesting that the dendrimers did not exert their neuroprotective effect interfering with NMDA receptor activation and that the protective effect was mediated by an intracellular action.

### 2.3. Phosphorous Dendrimers Decrease NMDA-Induced ROS Production

**Figure 3:**
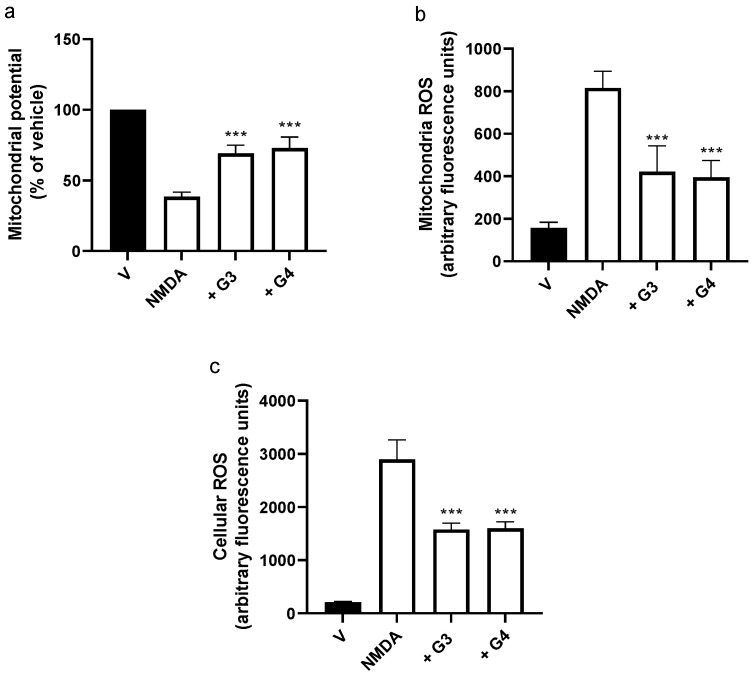
Effect of G3 and G4 phosphorous dendrimers on NMDA-induced changes in Ψm and ROS levels. Neurons were treated with vehicle (V), G3, or G4 phosphorous dendrimers (10 µM) for 1 h, and then NMDA (150 µM) was added. Neuronal (**a**) mitochondria potential, (**b**) mitochondrial ROS, and (**c**) total cellular ROS were determined as indicated in the Materials and Methods section. The data are expressed as the mean + the s.e.m. of 30 to 40 neurons obtained from four independent experiments. *** *p* < 0.001 as compared to NMDA-treated cells in the absence of dendrimers.

Next, we explored if the phosphorous dendrimers were acting at the mitochondrial level to prevent excitotoxicity. NMDA-induced Ca^2+^ overload has been related to a decrease in Ψm leading to an increase in ROS production and the activation of the intrinsic apoptotic pathway [32]. Exposure of mouse cortical neurons to NMDA (150 µM) induced a fast decrease in Ψm to about 40% of control values at 1 h (Figure S3a), which was followed by an increase in both mitochondrial (Figure S3b) and total ROS (Figure S3c) production. Both phosphorous dendrimer generations, G3 and G4, were able to partially prevent NMDA-induced decrease in Ψm (Figure 3a) and, accordingly, almost halved NMDA-induced mitochondrial (Figure 3b) and total ROS (Figure 3c) production. One possible explanation is that phosphorous dendrimers would be acting as antioxidant agents by either inhibiting ROS production or scavenging the produced ROS. As indicated in Figure S4, this is not the case since the generation of phosphorous dendrimers did not inhibit xanthine-oxidase-mediated superoxide generation either in a cell-free system or in lysates of both cortical neurons and brain organoids, while the superoxide dismutase (SOD) cell-permeant mimetic Mn(III)tetrakis (4-benzoic acid)porphyrin chloride (MnTABP) inhibited by more than 50% superoxide production (Figure S4).

### 2.4. Phosphorous Dendrimers Inhibit NMDA-Induced Intrinsic Apoptotic Pathway Activation

**Figure 4:**
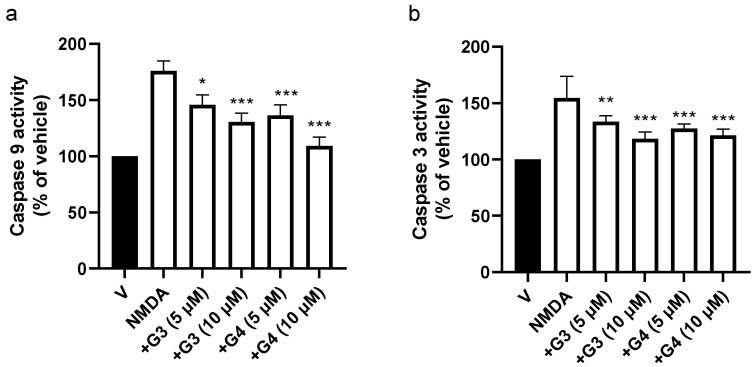
Effect of G3 and G4 phosphorous dendrimers on NMDA-induced (**a**) caspase 9 and (**b**) caspase 3 activities. Neurons were treated with vehicle (V) or the indicated concentration of G3 or G4 phosphorous dendrimers. Then, NMDA (150 µM) was added and incubation continued for 3 h. Caspase activity was determined in total lysates as described in the Materials and Methods section. The data are expressed as the mean + the s.e.m. of three independent experiments. * *p* < 0.05, ** *p* < 0.01, and *** *p* < 0.001 as compared to NMDA-treated cells in the absence of dendrimers.

Loss of Ψm has been related to caspase activation and induction of the intrinsic apoptotic pathway [33]. Thus, we studied the effect of phosphorous dendrimers on NMDA-mediated activation of caspases 9 and 3. Treatment of mouse cortical neurons with NMDA resulted in an early activation of caspase 9, coinciding with the loss of mitochondrial membrane potential (Figure S5a). Caspase 9 activation was followed by caspase 3 activation, which reached maximal activity at 3 h (Figure S5b). Lengths of time for maximal NMDA-induced caspase 3 and 9 activation were selected for the next experiments. Both phosphorous dendrimer generations reduced NMDA-induced caspase 9 (Figure 4a) and 3 (Figure 4b) enzymatic activity in a dose-dependent manner. This suggests that, at least in part, the neuroprotective effect of these phosphorous dendrimers was related to an inhibition of the intrinsic apoptotic pathway.

### 2.5. Phosphorous Dendrimers Inhibit NMDA-Induced Activation of the ER Stress Pathway

**Figure 5:**
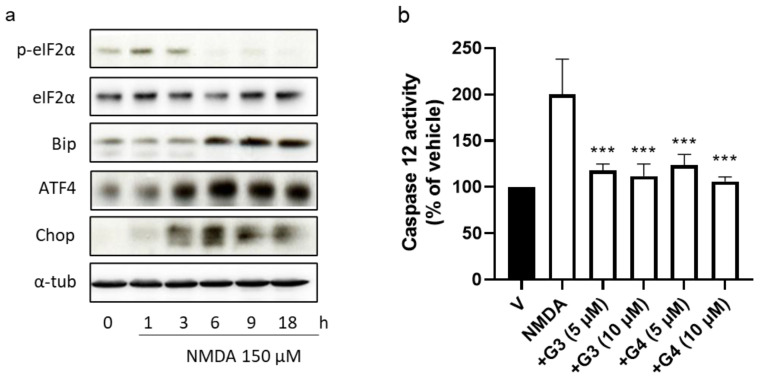
Effect of phosphorous dendrimers on NMDA-induced changes in ER-stress-signaling proteins and in caspase 12 activity. (**a**) Neurons were treated with vehicle (V) or the indicated concentration of G3 or G4 phosphorous dendrimers for 1 h. Then, NMDA (150 µM) was added and the incubation continued for another 1 h for p-eiF2α and eiF2α determination and for 6 h for Bip, ATF4, and Chop protein determination by Western blot, as described in the Materials and Methods section. α-Tubulin was used as loading control. The image shows one representative experiment that was repeated three times with similar results. (**b**) Neurons were treated with vehicle (V) or the indicated concentration of phosphorous dendrimers for 1 h. Then, NMDA (150 µM) was added and after 6 h, caspase 12 activity measured as indicated in the Methods section. The data represent the mean + the s.e.m. of three experiments. *** *p* < 0.001 as compared to NMDA-treated cells in the absence of dendrimers.

**Figure 6:**
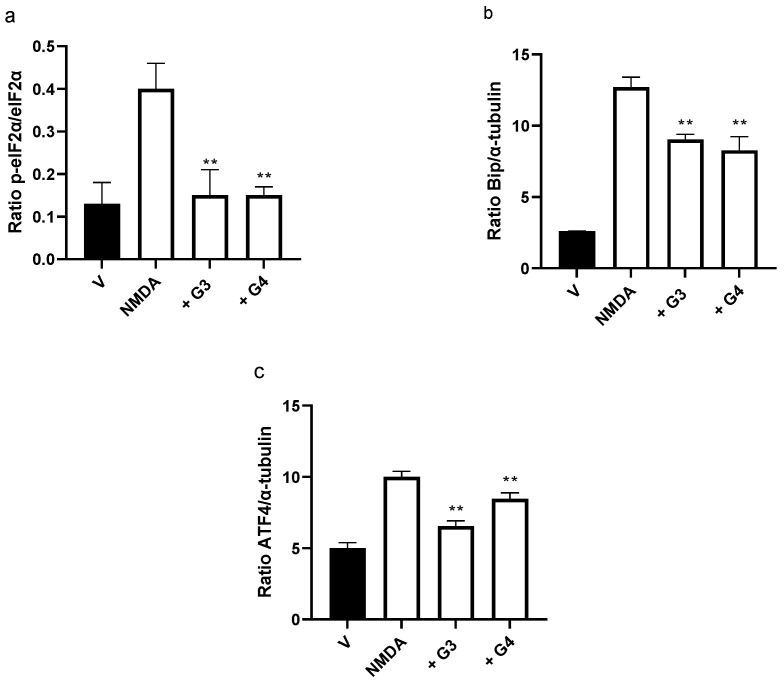
Quantification of the effect of phosphorous dendrimers on NMDA-induced changes in ER stress proteins. Neurons were treated with vehicle (V) or with 10 µM of G3 or G4 phosphorous dendrimers for 1 h. Then, NMDA (150 µM) was added and incubation continued for another 1 h for (**a**) p-eiF2α and eiF2α determination and for 6 h for (**b**) Bip and (**c**) ATF4 determination. α-Tubulin was used as loading control. The data are expressed as the mean + the s.e.m. of three experiments. ** *p* < 0.01 as compared to NMDA-treated cells in the absence of dendrimers.

Endoplasmic reticulum stress has been linked to excitotoxic neuronal death in several neurodegenerative disorders since it promotes mitochondrial dysfunction and induces specific ER stress and apoptosis pathways [29]. Next, we decided to explore whether phosphorus dendrimers were interfering with the ER stress response. First, we observed that NMDA (150 µM) induced the activation of the ER stress response, characterized by an initial and transitory increase in eIF2α phosphorylation (peIF2α), without modifying total levels of eIF2α total protein (Figure S6a; Table S1). In addition, increases in Bip, ATF4, and Chop proteins were observed at longer lengths of time (Figure S6a; Table S1), consistent with an ER stress response activation. Both generations of phosphorous dendrimers prevented NMDA-induced increase in peIF2α phosphorylation (Figure 5a and Figure 6a) and significantly reduced NMDA-induced increase in Bip (Figure 5a and Figure 6b) and CHOP (Figure 5a and Figure 6c) protein levels. NMDA also induced caspase 12 activation peaking at 6 h (Figure S5b), which was almost completely blocked by G3 and G4 phosphorous dendrimers (Figure 6b). As can be observed, these data strongly suggest ER stress and UPR response as possible targets for this direct neuroprotective action of neutral phosphorous dendrimers.

### 2.6. Phosphorous Dendrimers Inhibit NMDA-Induced Excitotoxicity in Brain Organoids

**Figure 7:**
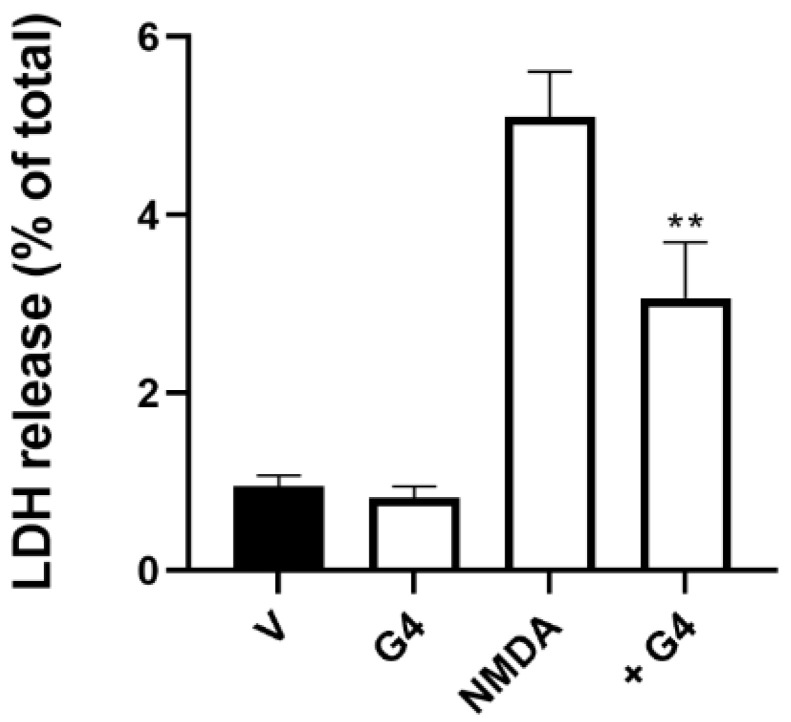
Phosphorous dendrimers prevent NMDA-mediated excitotoxicity in human brain organoids. Brain organoids were treated with either vehicle (V) or G4 phosphorous dendrimers (10 µM) for 1 h, and then NMDA (150 µM) was added and and incubation continued for another 24 h. LDH was determined as indicated in the Materials and Methods section. The data represent the mean ± the s.e.m. of six experiments. ** *p* < 0.01 when compared to NMDA-treated neurons in the absence of dendrimers.

Exposure of brain organoids to NMDA (150 µM; 24 h) induced the death of about 5% of the brain organoid neurons, this effect being markedly decreased by treatment of the organoids with G4 phosphorus dendrimers (10 µM) (Figure 7).

**Figure 8:**
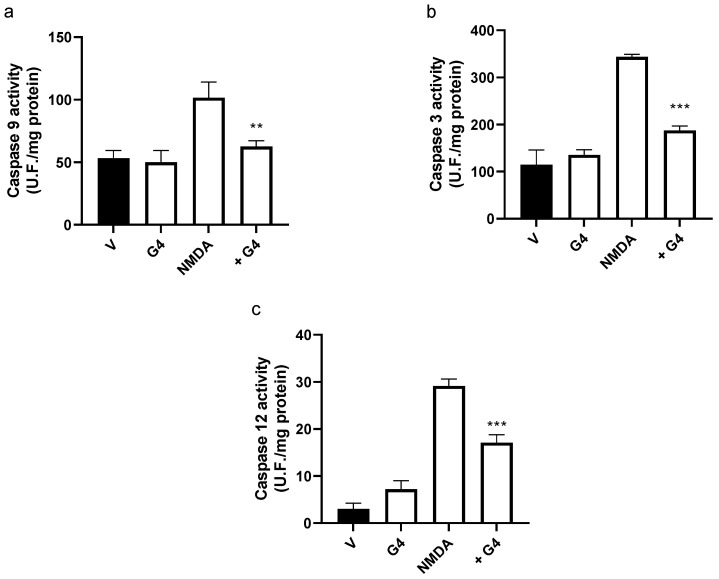
Effect of the phosphorous dendrimer G4 on NMDA-induced (**a**) caspase 9, (**b**) caspase 3, and (**c**) caspase 12 enzymatic activity in brain organoids. Brain organoids were treated with either vehicle (V) or G4 phosphorous dendrimer (10 µM) for 1 h, and then NMDA (150 µM) was added. Caspase 3 and 9 activities were determined at 3 h and caspase 12 at 6 h. Caspase activity was determined in total lysates as described in the Materials and Methods section. The data are expressed as the mean + the s.e.m. of five independent experiments. ** *p* < 0.01, *** *p* < 0.001 as compared to NMDA-treated cells in the absence of dendrimers.

In addition, the treatment of brain organoids with NMDA (150 µM; 24 h) induced a marked increase in caspases 9 (Figure 8a) and 3 (Figure 8b) enzymatic activities, suggesting activation of the intrinsic apoptotic pathway. Treatment of the organoid with G4 phosphorous dendrimer did not show any toxic effect. However, it markedly blocked NMDA-induced caspase activation in a similar way to the effect found in isolated cultured mouse cortical neurons (Figure 8a,b). NMDA also induced an increase in caspase 12 activity, suggesting the involvement of the ER stress response (Figure 8c). This activation was also decreased in the presence of G4 phosphorous dendrimers (Figure 8c).

## 3. Discussion

Nanoparticles are becoming increasingly involved in therapeutics as drug and genetic material delivery systems [2,3]. This is due to the fact that they can be synthesized with a high degree of control of the structural parameters, yielding highly monodisperse chemical structures [34]. However, nanoparticles can themselves show therapeutic properties in the absence of other added drugs or genetic material [12]. Dendrimers are a polymeric type of nanoparticles that have a promising role in therapeutics [14]. Among the different types of dendrimers, phosphorous dendrimers possess interesting properties for imaging, the delivery of nucleic acids or drugs, and as drugs by themselves. Cationic phosphorous dendrimers have shown activity against scrapie, preventing protein aggregation of prions, responsible for the development of transmissible spongiform encephalopathies [13]. Moreover, neutral phosphorous dendrimers present anti-inflammatory properties both in vitro and in vivo [12].

Excitotoxicity plays a key role in the pathogenesis of several CNS diseases, including stroke [35] and Alzheimer’s [36] and Parkinson’s [37] diseases. Excitotoxicity is caused by over-stimulation by glutamate of ionotropic receptors NMDA, α-amino-3-hydroxy-5-methyl-4-isoxazolpropionic acid (AMPA), and Kainate [23]. Excessive activation of the ionotropic receptors, mainly NMDAR, induces a massive Ca^2+^ influx into the cells, leading to neuronal death in the CNS [38]. Besides intensive research efforts, no effective treatment is available for these diseases yet, indicating the need for new therapeutic agents.

Excitotoxicity-induced increase in [Ca^2+^]_i_ leads to mitochondrial dysfunction, an increase in ROS, and activation of several proinflammatory molecules, including NF-κB, which causes neuroinflammation. All these mechanisms contribute to neurodegeneration [39]. Since the aza-bisphosphonate-terminated neutral phosphorous dendrimers used in this work have shown anti-inflammatory properties both in vitro and in vivo by preventing NF-κB translocation to the cell nucleus [12], and neuroinflammation has been proposed as one of the possible mechanisms contributing to the pathogenesis of Alzheimer’s disease [40], we decided to study whether these neutral phosphorous dendrimers were able to inhibit neuronal excitotoxic death.

The phosphorous dendrimers did not interfere with NMDA-mediated Ca^2+^ influx since [Ca^2+^]_i_ in response to NMDA were similar in the presence and absence of phosphorous dendrimers. This excludes the fact that the nanoparticles will behave as NMDA receptor blockers and strongly suggests an intracellular mechanism of action, which has an important implication: neutral phosphorous dendrimers are able to gain access to the neuron interior, which is quite uncommon for an NP. This opens up a wide range of possibilities for their use in therapeutics of nervous system diseases.

Marked Ca^2+^ influx following NMDAR stimulation leads to mitochondrial Ca^2+^ overload, which results in the formation of a non-selective pore across the mitochondrial membrane, known as the mitochondrial permeability transition pore [41]. The pore collapses Ψm; increases ROS production [42]; and promotes the release of pro-apoptotic molecules; such as cytochrome c and Apaf-1 [43], which, in turn, will sequentially activate caspases 9 and 3, the latter being an executioner caspase whose activation results in cellular apoptosis [44]. Both G3 and G4 phosphorous dendrimers significantly inhibit NMDA-induced Ψm collapse and ROS production, both locally in the mitochondria and in the whole neuron, and induction of caspase 9 and 3 activities. However, antioxidant compounds have shown neuroprotection in vitro [45,46] and one possible mechanism involved in the neuroprotective action of phosphorous dendrimers on NMDA-mediated excitotoxicity could be to interfere with ROS production in a step beyond Ψm in addition to their action diminishing NMDA-mediated Ψm collapse. This does not seem to be the case, since in cell-free systems or in cortical neurons or brain organoid lysates, neither did phosphorous dendrimers show any effect on xanthine oxidase enzymatic activity nor did they act as a ROS scavenging compound, while the SOD mimetic MnTABP, used as the control, did. These results suggest that one of the possible mechanisms involved in the neuroprotective action of neutral phosphorus dendrimers might be to interfere with NMDA-induced Ψm collapse and thus with the intrinsic-apoptotic-signaling pathway.

However, Ca^2+^ buffering is one of the processes that link mitochondria and ER functions through the formation of functional structures called mitochondrial-associated membranes [47]. The connection between ER and mitochondria regulates ATP production, mitochondrial dynamics, lipid biosynthesis, and cell survival [48]. Therefore, mitochondrial dysfunction triggered by excitotoxicity could also alter ER function, leading to UPR activation. In fact, we have shown that NMDA-induced activation of the ER stress response was characterized by a rapid increase in the phosphorylation of eIF2α, which was followed by the up-regulation of the chaperonin GRP-78/Bip, ATF4, and CHOP. It is noteworthy that ATF4 and its downstream target, CHOP, are both transcription factors involved in ER-stress-induced apoptosis [49]. An increase in caspase 12 activity was also detected at later times, suggesting that it might also contribute to NMDA-induced neuronal death [50]. Thus, it would be possible that another target for phosphorous dendrimers to inhibit NMDA-mediated excitotoxic death was ER stress. This is the case since both G3 and G4 phosphorus dendrimers were able to completely block eIF2α phosphorylation and to partially inhibit ER-stress-induced expression of Bip and ATF4 as well as caspase 12 activity.

Brain organoids represent a marked step forward in terms of transability of basic research results to the clinical setting for several diseases, including neurodegenerative diseases. Organoids recapitulate several of the characteristics of the human brain, such as 3D organization achieved through self-organization of the developing organoids, making them an attractive model for the human brain by maintaining mechanical and cellular topographical relationships that generate close-to-physiological microenvironments [51]. This makes them more useful in providing information about the effect of possible therapeutic compounds on the specific human-pathogenic-signaling cascades involved in neuronal death than two-dimensional neuronal culture or animal models of neurologic disease [52]. Here, we show that NMDA induces excitotoxic death in human brain organoids. It is important to note that the percentage of NMDA-induced neuronal death, as indicated by the release of LDH to the medium, is markedly lower in the case of brain organoids than for isolated cultured mouse neurons. This indicates the more complex nature of brain organoids comprising different cell types, many of them lacking NMDA receptors, as it happens in the human brain, and also a complex tridimensional organization, while isolated cultured neurons constitute a homogeneous cell population containing NMDA receptors. Moreover, NMDA induced, in brain organoids, an increase in the activity of caspases 3 and 9, biochemical markers of the intrinsic apoptotic pathway. In addition, NMDA induced an increase in caspase 12 activity, which occurs mainly in response to ER stress. These data indicate that NMDA activates in the brain organoid the same mechanisms of excitotoxic death as it does in cultured neurons. It is important to note that G4-biocompatible bisphosphonate-terminated neutral phosphorous dendrimers were able to markedly reduce these deleterious actions of NMDA in brain organoids, indicating that the dendrimer not only is able to penetrate the 3D structure of the brain organoid to reach the cortical plate area but also faithfully reproduces its pharmacological actions on NMDA-induced excitotoxic death observed in primary neuronal cultures.

## 4. Materials and Methods

### 4.1. Chemical Synthesis

The synthesis of phosphorous dendrimers, generations 3 and 4, bearing 48 and 96 terminal bisphosphonate groups, respectively, was performed as previously described [12].

### 4.2. Animals

All experimental procedures were conducted and animal care performed in accordance with the guidelines of the Ethical Committee of Animal Experimentation (CEEA), which issued the proper permit (protocol number PR-2017-07-18, approved July 18, 2017) at the University of Castilla-La Mancha (UCLM), in accordance with the guidelines of the European Union (2010/63/EU) for the use of laboratory animals, and the ARRIVE guidelines were followed for experiments involving animals [53].

### 4.3. Cell Culture

Primary cultures of brain cortical neurons were obtained as previously described [54]. Briefly, fronto-lateral cortical lobes from E17 C57BL/6 mice fetuses were dissected and the cells were chemically dissociated in the presence of trypsin and DNAse I. The isolated cells were resuspended in serum-free Neurobasal^®^ medium supplemented with B27 containing penicillin (20 units mL^−1^), streptomycin (5 µg mL^−1^), and 2 µM L-glutamine. Cells were plated on poly-L-lysine-coated 24- or 6-well culture plates or on poly-L-lysine-coated glass coverslips and maintained at 37 °C in a saturated atmosphere containing 95% air and 5% CO_2_. Cortical neurons were used for experiments after being cultured for 7–12 days in vitro (DIV).

### 4.4. Brain Organoids

Brain organoids were generated following the procedure previously described [15]. Briefly, human pluripotent stem cells (PSCs; ATCC, Barcelona, Spain) were allowed to generate embryonic bodies that were induced to differentiate to neural tissue in a medium with low content of b Fibroblast Growth Factor (bFGF) and a high concentration of Rho-associated coiled-coil containing protein kinases (ROCK) inhibitor. Embryonic bodies were placed in Matrigel and allowed to generate a properly oriented neuroepithelium and self-organized 3D structure under continuous agitation as previously described [55].

### 4.5. Cellular Toxicity

At DIV 7–12, cortical neurons were treated with either vehicle (double-distillated water; ddH_2_O) or NMDA (150 µM) in the absence or presence of G3 or G4 phosphorous dendrimers at different concentrations. Twenty-four hours later, supernatants were collected and the cells washed with phosphate-buffered saline (PBS) and lysed with 0.9% Triton X-100 (V/V) in saline. Lactate dehydrogenase (LDH) activity release to the culture medium was used as an indicator of cellular death [56]. LDH release to the medium as well as that present in the cell lysates were determined using the Cytotox 96 Kit (Promega, Madison, WI, USA) following the manufacturer’s instructions. Cell mortality was expressed as the percentage of LDH released with respect to the total LDH content in the cells at the beginning of treatment.

### 4.6. Intracellular Ca^2+^ Measurement

Cytosolic Ca^2+^ was determined as previously described [11]. Cortical neurons (DIV 7–12) were seeded on poly-L-lysine-coated glass coverslips (20 mm) and treated with either vehicle or G3 or G4 phosphorous dendrimers (10 µM; 1 h). Afterward, cells were incubated in Krebs–Henseleit (K–H) solution with the ionic composition (in mM) NaCl, 140; CaCl_2_, 2.5; MgCl_2,_ 1; KCl, 5; Hepes, 5; and Glucose, 11 (pH, 7.4) solution containing Fura-2 (5 µM) and 0.005% (*v*/*v*) Pluronic (Thermo Fisher, Madrid, Spain) for 30 min at 37 °C in the dark. Coverslips were washed twice with K–H solution and mounted on the stage of a Nikon Eclipse TE2000-E fluorescence microscope (Nikkon, Tokyo, Japan). The cells were excited alternately at 340 nm and 380 nm and emitted fluorescence recorded at 510 nm using a CCD camera (Hamamatsu Photonics, Shizuoka, Japan), at 200 Hz sampling rate, using the NIS Elements AR software (Nikkon, Tokyo, Japan). Basal [Ca^2+^]_i_ was obtained for the initial 39 s, and then NMDA (150 µM) was added and frames recorded for 60 additional seconds.

### 4.7. ROS Generation

Cortical neurons (DIV 7-12) were treated with vehicle or NMDA (150 µM) in the absence or presence of G3 or G4 phosphorous dendrimers at the indicated concentrations for different lengths of time. Afterward, the cells were incubated in K–H solution containing the ROS-sensitive fluorescent dye chloro-methyl 2′,7′-dichlorodihydrofluorescein diacetate (CM-H2DCFDA 10 µM; Molecular Probes, Barcelona, Spain) or with the mitochondrial-specific superoxide-sensitive fluorescent dye MitoSOX Red (2.5 µM; Invitrogen, Carlsbad, CA, USA) for 30 min at 37 °C. After being washed twice with K–H solution, the coverslips were placed on the stage of a Nikon Eclipse TE2000-E fluorescence microscope (Nikon, Tokyo, Japan). Excitation and emission wavelengths were set at 535 nm and 635 nm for H2DCFDA fluorescence and 510 nm and 580 nm for MitoSOX Red, respectively. Samples were recorded every 15 s using a CCD camera (Hamamatsu Photonics, Shizuoka, Japan) and analyzed using the NIS Elements AR software (Nikkon, Tokyo, Japan). Recorded fluorescence for each experimental condition was fitted to the equation y = a + bx, and the slope b was taken as an index of the rate of superoxide production as previously described [57].

### 4.8. Mitochondrial Transmembrane Potential

The mitochondrial transmembrane potential (Ψm) was determined as previously described [58]. Briefly, cortical neurons (DIV 7-12) were treated with either vehicle or NMDA (150 µM) in the absence or presence of G3 or G4 phosphorous dendrimers at the indicated concentrations for different lengths of time. Afterward, the cells were incubated in K–H solution containing tetramethylrhodamine methyl ester (10 µM; TMRM) (Thermo Fisher, Madrid, Spain), washed with K–H solution, and mounted on the stage of a Nikon Eclipse TE2000-E fluorescence microscope (Nikon, Tokyo, Japan). The cells were excited at 535 nm wavelength and emitted fluorescence recorded at 590 nm using the NIS Elements AR software (Nikkon, Tokyo, Japan). Samples were recorded every 15 s for 5 min with a CCD camera (Hamamatsu Photonics, Shizuoka, Japan). Decaying fluorescence signal showing mitochondrial potential was fitted using a linear regression model and the least squares method. The slopes of the fitted lines were taken as the rate of loss of Ψm. Percentages of Ψm loss rates were calculated with respect to vehicle-treated cells.

### 4.9. Extraction of Total Lysates

Cortical neurons (DIV 7-12) were treated with either or NMDA (150 µM) in the absence or presence of G3 or G4 phosphorous dendrimers at the indicated concentrations for different lengths of time. Afterward, the cells were washed twice with cold PBS and resuspended in homogenization buffer (10 mM Hepes, 0.32 M sucrose, 100 µM EDTA, 1 mM DTT, 0.1 mM phenylmethylsulfonyl fluoride (PMSF), 40 µg mL^−1^ aprotinine, and 20 µg mL^−1^ leupeptine; pH 7.4). Cortical neurons were homogenized using a polytron (two cycles; 10 s at maximum speed). Afterward, the homogenates were centrifuged at 10,000× *g* and supernatants (i.e., total lysates) collected and stored at −80 °C until analysis by gel electrophoresis or caspase activity determination was performed.

### 4.10. Caspase Activities Determination

Caspase 3, caspase 9, and caspase 12 activities were determined in total lysates as previously described [56]. Briefly, fluorescent substrates Z-Asp-Glu-Val-Asp-7-Amino-4-trifluoromethylcoumarin (Z-DEVD-AFC), Ac-Leu-Glu-His-Asp-7-Amino-4-trifluoromethylcoumarin (Ac-LEHD-AFC), and Ac-Ala-Thr-Ala-Asp-7-Amino-4-trifluoromethylcoumarin (Ac-ATAD-AFC) were used to determine caspase 3, 9, and 12 activities, respectively. Lysates (50 µg of protein) were incubated at 37 °C for 1 h following the manufacturer’s instructions (BioVision, Milpitas, CA, USA). Cleavage of the AFC fluorophore was determined in a spectrofluorometer (Victor3; Perkin Elmer, Madrid, Spain) at an excitation wavelength of 400 nm and at an emission wavelength of 505 nm. Caspase activities were calculated as units of fluorescence formed per milligram of protein per hour.

### 4.11. Western Blot Assay

Immunoblot analysis was performed on total lysates as previously described [54]. Protein samples from total lysates (25 μg) were loaded onto 10% or 15% PAGE-SDS gels and transferred to nitrocellulose membranes. Membranes were blocked in PBS-Tween 20 (0.1%) containing 5% non-fat dry milk and 0.1% BSA for 1 h at 4 °C and incubated with primary antibodies directed against the protein of interest overnight at 4 °C. The primary antibodies used were rabbit monoclonal anti-eIF2α antibody (1:1000), rabbit monoclonal anti-peIF2α antibody (1:1000), rabbit monoclonal anti-Bip antibody (1:1000), and rabbit monoclonal anti-ATF4 antibody (1:1000), all of them obtained from Cell Signaling (Leiden, The Netherlands); mouse monoclonal anti-CHOP antibody (1:1000) (RD systems, Minneapolis, MN, USA); and mouse polyclonal α-tubulin antibody (1:2000) (Calbiochem, Barcelona, Spain). Afterward, the membranes were washed and incubated with the corresponding secondary antibody HRP anti-rabbit IgG (1:10,000) or HRP anti-mouse IgG (1:10,000). Immunoreactive bands were visualized using an enhanced chemiluminescence system (ECL, Amersham BiosciencesGE Healthcare, Upsala, Sweden). Densitometric analysis of immunoreactive bands was performed using ImageQuant 5.2 software (GE Healthcare, Uppsala, Sweden).

## 5. Conclusions

In summary, both G3- and G4-biocompatible bisphosphonate-terminated neutral phosphorus dendrimers showed strong neuroprotective actions against NMDA-induced excitotoxic neuronal death in primary neuronal cultures. These actions are not related to the inhibition of NMDA-mediated Ca^2+^ influx but to an intracellular mechanism of action. It is important to note that it is not easy for NPs, either alone or carrying a cargo, to enter the neurons. These two phosphorous dendrimers are one of the few examples of NPs entering primary neuronal cultures, which opens a wide range of possibilities to use these NPs to study neuronal physiology and pathophysiology. In addition, these neutral phosphorous dendrimers can interfere with excitotoxic mechanisms at two different levels: preventing Ψm collapse and reducing the UPR response that follows ER stress. Moreover, these phosphorous dendrimers can penetrate human brain organoids, where they can interfere with NMDA-induced mechanisms of neuronal death in a similar way as they do in isolated neuronal cultures, indicating the ability of the dendrimers to penetrate complex tridimensional structures, such as brain organoid structures, which replicate a significant number of properties of the human brain. Taken together, these actions, combined with the anti-inflammatory properties previously shown in vivo and in vitro [12], strongly suggest that these NPs have the potential to be used as scaffolds to generate new macromolecules as compounds that might be effective for neurodegenerative diseases treatment. This approach might open new avenues for the treatment of neurodegenerative diseases.

## Data Availability

The manuscript data can be made available from the corresponding author following reasonable request.

## Supplementary Materials

The following supporting information can be downloaded at: https://www.mdpi.com/article/10.3390/ijms23084391/s1. Reference [59] is cited in Supplementary Materials.
