# Supplementary material for: Engineered Neutral Phosphorous Dendrimers Protect Mouse Cortical Neurons and Brain Organoids from Excitotoxic Death
Source: Int J Mol Sci. 2022 Apr 15;23(8):4391. doi: 10.3390/ijms23084391 (PMC9024777; doi:10.3390/ijms23084391)
Supplement: Supplementary file 1 [file ijms-23-04391-s001.zip › ijms-1652377-supplementary.pdf]

Supplementary Materials

Methods

Superoxide anions were also generated by the hypoxanthine/xanthine oxidase system as previously described [59]. Briefly, the reaction mixture contained 50 mM KH_2PO_4-KOH (pH 7.4), 1 mM EDTA, and 100 μM hypoxanthine and nitroblue tetrazolium chloride (NBT) (25 μM) in a total volume of 200 μL. The reaction was started by adding 0.2 mU of xanthine oxidase or 30 μg of cortical neurons or brain organoid total lysate. Superoxide radicals were measured by spectrophotometry at 560 nm wavelength 20 min after enzyme addition.

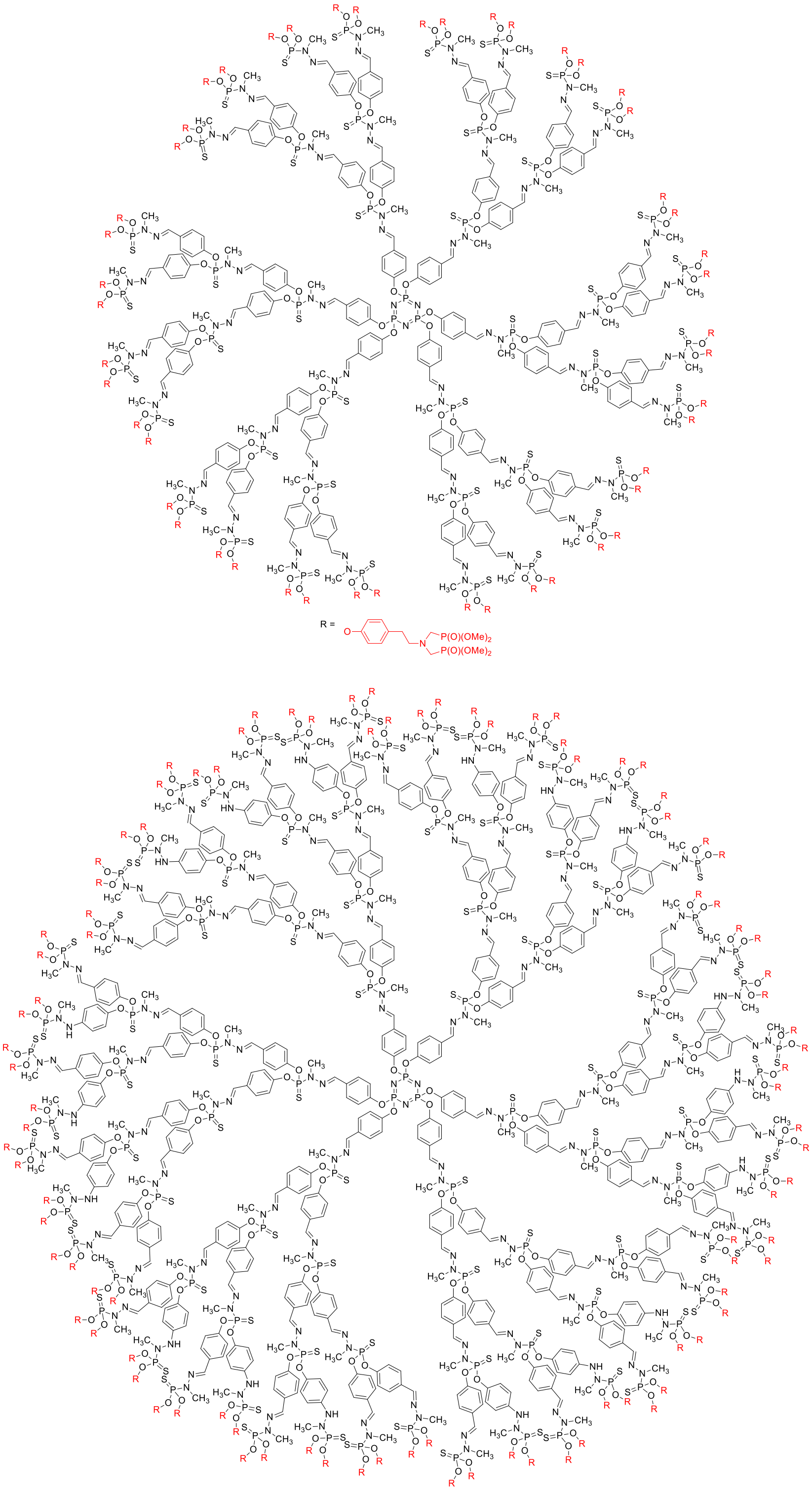

Figure S1. Structure of G3 (top) and G4 (bottom) phosphorous dendrimers.

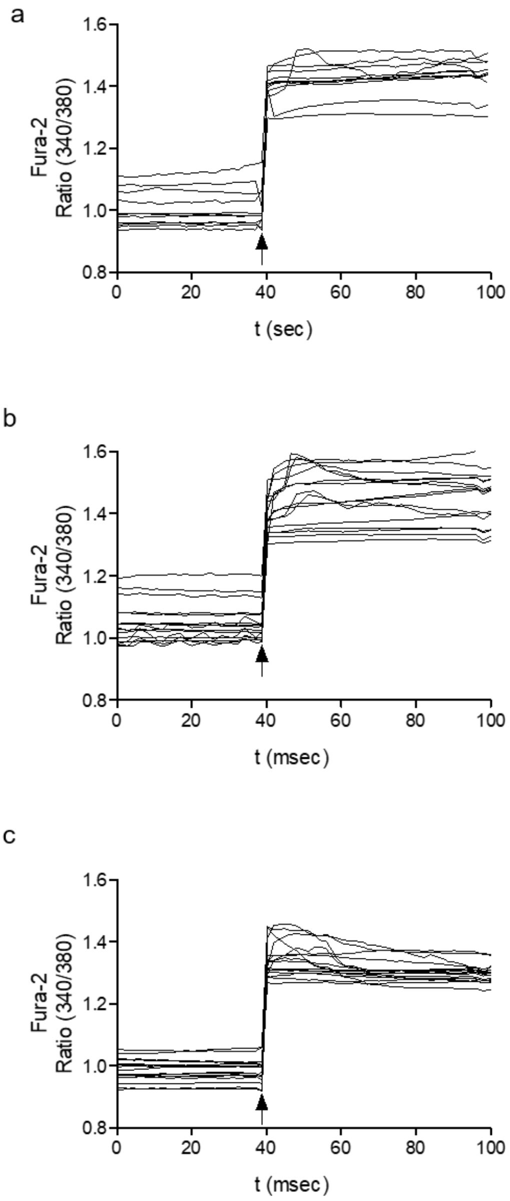

Figure S2. Representative records of NMDA-induced increase in [Ca^{2+}]$_i$ in cultured neurons. Neurons were incubated with Fura-2 as indicated in the Materials and Methods section. After 39 s of basal recording, NMDA (150 µM) was added (arrows) and recording continued for an additional 60 s. The figure shows representative experiments of NMDA-induced increase in [Ca^{2+}]$_i$ in (a) the absence or presence of (b) G3 (10 µM) or (c) G4 (10 µM) that were repeated three times with similar results.

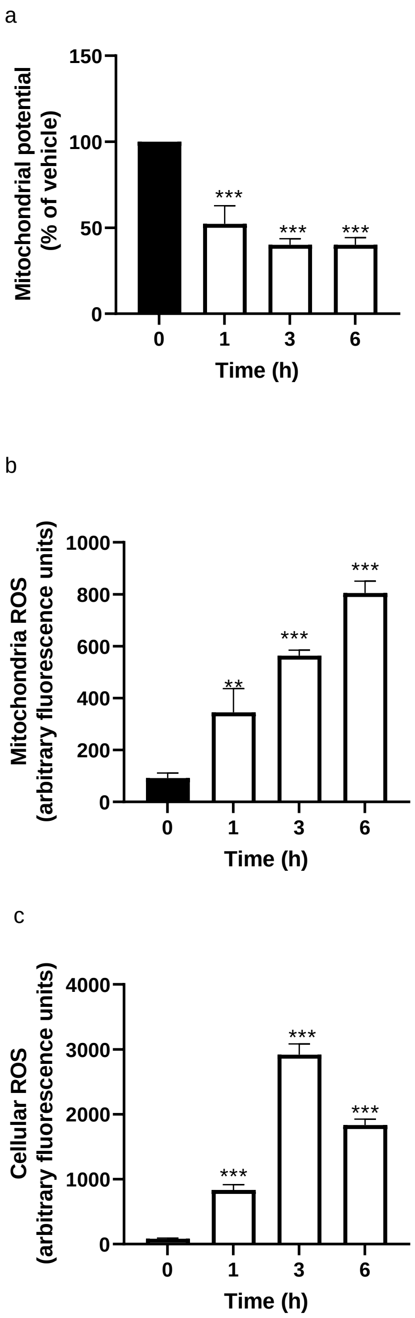

Figure S3. Time-course of NMDA-induced changes in Ψm and ROS levels. Neurons were incubated with (a) TMRM, (b) Mitosox, and (c) CM-H2DCFDA as indicated in the Materials and Methods section and exposed to NMDA (150 μM) for different lengths of time. Fluorescence was recorded as indicated in the Materials and Methods section. Data are represented as the mean ± the s.e.m. obtained from 30 to 40 neurons in four independent experiments for: (a) mitochondrial potential, (b) mitochondrial ROS, and (c) total neuronal ROS. **p < 0.01; ***p < 0.001 as compared to time 0 untreated cells.

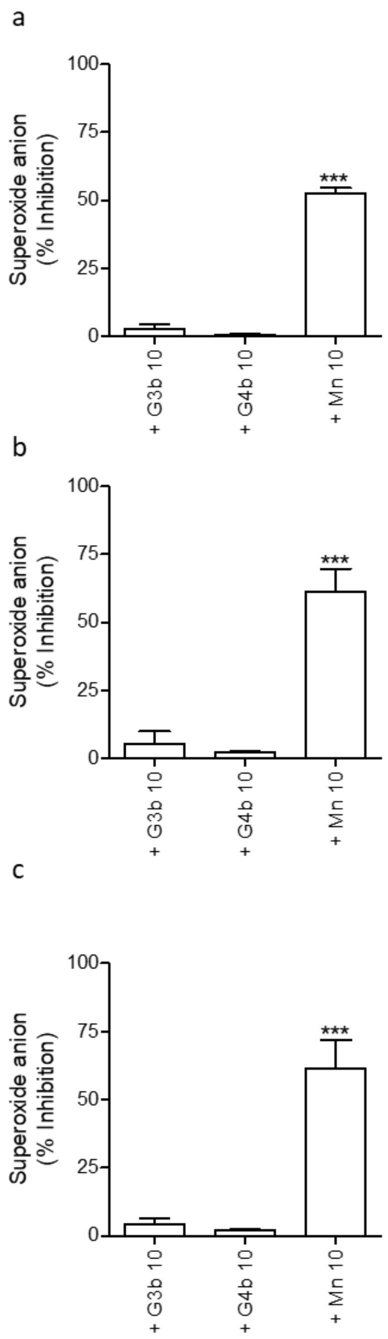

Figure S4. Effect of G3b, G4b, and MnTBAP on superoxide production in three different cell-free systems: (a) Commercial isolated xanthine oxidase, (b) total lysates from cortical neurons, and (c) total lysates from neuronal organoids. Lysates were used as an enzyme source to test the antioxidant activity of phosphorous dendrimers or MnTBAP as a reference compound. Data are the mean ± the s.e.m. of six independent experiments. ***$p < 0.001$ compared to control values in the absence of any compound.

a

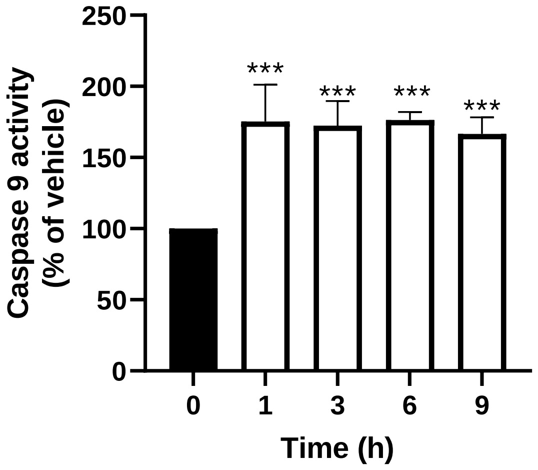

b

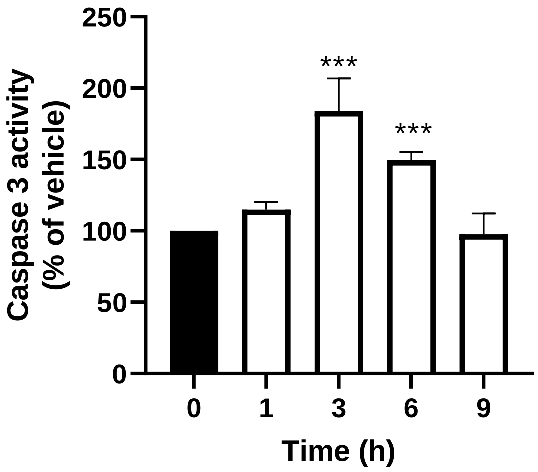

Figure S5. Time-course of NMDA-induced caspase 9 and 3 activities. Neurons were treated with NMDA (150 μM) for different lengths of time. Caspase activity was determined in total lysates as described in the Materials and Methods section. Data are expressed as the mean ± the s.e.m. of three independent experiments. ***p < 0.001 as compared to time 0 untreated cells.

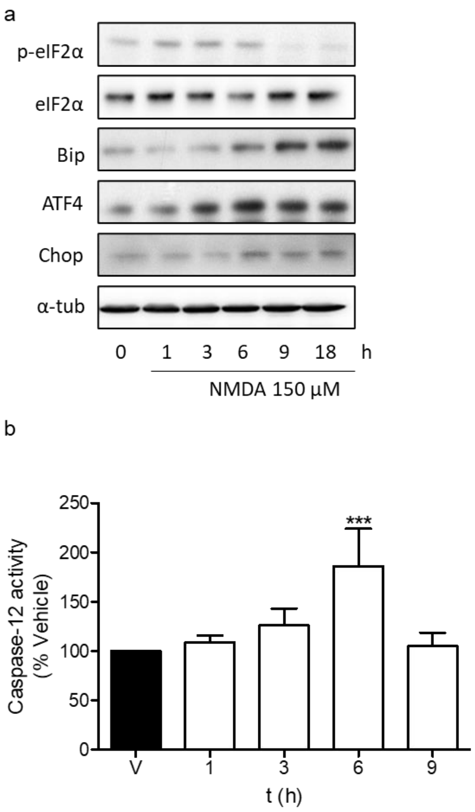

Figure S6. Time-course of NMDA-induced changes in ER-stress-signaling proteins and in caspase 12 activity. (a) Neurons were treated with NMDA (150 µM) for the indicated lengths of time and p-eiF2α, eiF2α, Bip, ATF4, and Chop protein amounts were determined by Western blot as described in the Materials and Methods section. α-Tubulin was used as the loading control. The image shows one representative experiment that was repeated three times with similar results. (b) Neurons were treated for 1 h with the indicated concentration of G3 or G4 phosphorous dendrimers. Then, NMDA (150 µM) was added, the mixture was left for 6 h, and caspase 12 activity was measured as indicated in the Materials and Methods section. Data represent the mean ± the s.e.m. from three experiments. ***$p < 0.001$ as compared to time 0 untreated cells.

Table S1. Time-course of NMDA-induced changes in ER-stress-related proteins in cortical neurons. Mouse cortical neurons were exposed to NMDA (150 μM) for the indicated lengths of time and the ratios of protein/α-tubulin, used as the loading control, were determined following densitometric analysis of the Western blot signals and referring to the ratios observed at time 0. Data represent the mean ± the s.e.m. of three experiments. (a) $p < 0.05$; (b) $p < 0.01$; (c) $p < 0.001$ when compared to time 0.

**Time (h)**	**0**	**1**	**3**	**6**	**9**	**18**
p-eIF2*α*	100	187±14[b]	124±12[a]	22±12	31±3	18±12
eIF2*α*	100	114±13	94±11	99±9	107±13	107±10
p-eIF2*α*/ eIF2*α*	100	142±7[b]	118±6[a]	26±2[b]	187±14[b]	187±14[b]
Bip	100	78±7	93±11	208±20[b]	260±17[c]	249±12[c]
ATF4	100	35±6	77±7	280±18[b]	478±30[c]	496±27[c]
CHOP	100	256±9[b]	720±27[c]	984±26[c]	733±24[c]	682±26[c]
